# Associations of eating behaviors, dietary inflammation, and visceral adiposity with clinical and psychological features in epilepsy

**DOI:** 10.1016/j.ebr.2026.100866

**Published:** 2026-04-09

**Authors:** Sinan Eliaçık, Sena Yanıkoğlu, Ayşe Erdoğan Kaya

**Affiliations:** a Hitit University School Of Medicine Department of Neurology, Turkey; b Hitit University School Of Medicine Department of Psychiatry, Turkey; c Kırıkkale University Nutrition and Dietary Department, Turkey

**Keywords:** Epilepsy, Dietary inflammatory index, Visceral adiposity index, Inflammation

## Abstract

**Objective:**

Systemic inflammation has been increasingly implicated in the pathogenesis of various chronic diseases, including epilepsy. This study aimed to investigate the associations between eating behaviors and clinical characteristics of epilepsy by evaluating the Dietary Inflammatory Index (DII), Visceral Adiposity Index (VAI), and the Three-Factor Eating Questionnaire.

**Methods:**

This single-center, cross-sectional study included a total of 290 participants, comprising 150 individuals diagnosed with epilepsy and 140 control subjects without epilepsy.

**Results:**

Compared with controls, individuals with epilepsy demonstrated significantly higher emotional and uncontrolled eating scores, whereas no significant difference was observed in cognitive restraint. Within the epilepsy group, emotional eating, uncontrolled eating, VAI, and seizure frequency were positively correlated with DII, while cognitive restraint showed a negative association. Higher DII scores were observed among patients receiving polytherapy and those experiencing frequent seizures. Additionally, women with epilepsy exhibited higher DII values, whereas men demonstrated higher VAI scores. Path analysis demonstrated that DII exerted both direct and indirect effects on seizure frequency, partially mediated by emotional eating and visceral adiposity.

**Conclusions:**

These findings indicate a close interplay between dietary behaviors, systemic inflammation, and epilepsy-related clinical features. The results emphasize the need for long-term, comprehensive studies focusing on inflammatory dietary patterns and their potential role in the management of epilepsy.

## Highlights

• Eating behaviors were associated with clinical and psychological features in epilepsy.
• Higher dietary inflammatory index scores were linked to greater seizure burden.
• Visceral adiposity showed a significant relationship with seizure frequency.
• Dietary inflammation may indirectly influence epilepsy severity via visceral fat.
• Nutritional and metabolic factors may play a role in epilepsy management.

## 1 Introduction

Adequate and balanced nutrition refers to the sufficient intake and effective utilization of essential nutrients required for growth, development, and the maintenance of body tissues. Eating behaviors are shaped by a multifactorial interaction of physiological, psychological, sociocultural, economic, and disease-related determinants [1], [2]. In the context of epilepsy, nutritional research has largely focused on therapeutic dietary interventions, most notably the ketogenic diet, particularly in pediatric populations. In contrast, habitual eating behaviors and their broader metabolic and inflammatory implications have received comparatively limited attention [3].

Psychological factors are among the key regulators of eating behavior. Emotional states such as stress, anxiety, or anger can substantially influence food intake, leading individuals to consume food either excessively or inadequately as a coping response to emotional distress [4], [5]. Emotional eating is defined as food consumption driven primarily by emotional cues rather than physiological hunger and is frequently associated with increased intake of energy-dense foods rich in sugar and fat [6]. Conversely, daily energy intake under normal conditions is regulated by biological hunger and satiety mechanisms, which are modulated by individual characteristics including age, sex, body composition, and metabolic rate [7].

Dietary composition also has important implications for systemic inflammation. The Dietary Inflammatory Index (DII) was developed based on an extensive body of scientific literature to quantify the inflammatory potential of an individual’s diet by evaluating the relationships between 45 dietary components and inflammation-related biomarkers. This index provides a comprehensive measure of the overall pro- or anti-inflammatory nature of dietary intake [8], [9], [10], [11].

In addition to dietary factors, body fat distribution is a major determinant of metabolic and inflammatory status. The Visceral Adiposity Index (VAI), introduced in 2010, is a sex-specific marker designed to estimate adipose tissue dysfunction by integrating anthropometric and metabolic parameters and is widely used as an indirect indicator of cardiometabolic risk [12]. In individuals with epilepsy, disease-related restrictions, long-term use of antiseizure medications, and lifestyle-related factors may contribute to unfavorable alterations in body composition, including increased visceral fat accumulation.

Given the complex interrelationships among eating behaviors, dietary inflammatory load, visceral adiposity, and psychological factors, a comprehensive evaluation of these variables in individuals with epilepsy is warranted. Therefore, the present study aimed to investigate eating behaviors, dietary inflammatory potential as assessed by the DII, visceral adiposity as estimated by the VAI, and their associations with depression, anxiety, and selected epilepsy-related clinical characteristics in individuals with epilepsy.

## 2 Statistical analysis

All statistical analyses were performed using SPSS software version 24.0. Continuous variables were assessed for normality and are presented as mean ± standard deviation when normally distributed. Normality was evaluated using the Shapiro–Wilk test. Comparisons between two independent groups were conducted using the Student’s t-test for normally distributed variables, whereas the Mann–Whitney U test was applied for variables that did not meet normality assumptions. Comparisons involving three or more groups were performed using the Kruskal–Wallis test. Associations between categorical variables were examined using the chi-square test. A two-tailed p value of less than 0.05 was considered statistically significant. In addition, path analysis was performed to examine the direct and indirect relationships among dietary inflammatory load, psychological factors, visceral adiposity, and seizure frequency. The model was estimated using the maximum likelihood method and indirect effects were tested using bootstrap resampling with 5000 samples. Model fit was evaluated using χ^2^/df, CFI, TLI, RMSEA, and SRMR indices.

## 3 Materials and methods

The study was observational and non-interventional in nature. All procedures were conducted in accordance with the Declaration of Helsinki. Written informed consent was obtained from all participants. According to local regulations, formal ethics committee approval was not required for questionnaire-based observational studies. This was a single-center cross-sectional observational study conducted between January and October 2025. Participants were eligible for inclusion if they were between 18 and 55 years of age, had a confirmed diagnosis of idiopathic generalized epilepsy, and were able to communicate effectively and comply with study procedures. Female participants were included only if they were neither pregnant nor breastfeeding at the time of enrollment. Participants with a history of diabetes mellitus, cardiovascular disease, or other significant metabolic disorders were excluded from the study to minimize potential confounding effects on dietary inflammatory load and visceral adiposity. Individuals diagnosed with secondary (symptomatic) epilepsy were excluded from the study. The control group consisted of healthy volunteers recruited from the same geographical region during the same period. To minimize potential selection bias, controls were selected from individuals without a history of epilepsy or chronic neurological disorders and were matched with the epilepsy group in terms of age, sex, and educational status. Seizure frequency was categorized as once or twice per year, once or twice per month, and once or twice per week based on patient self-reports during the clinical interview.

Data collection was carried out through face-to-face interviews. Demographic characteristics were recorded, followed by anthropometric and biochemical measurements used to calculate the Visceral Adiposity Index (VAI) according to established sex-specific equations [13], incorporating body mass index (BMI), waist circumference, triglyceride levels, and high-density lipoprotein cholesterol (HDL-C):

Men:VAI = [Waist circumference (cm) / (36.58 + (1.88 × BMI))] × (Triglycerides / 1.03) × (1.31 / HDL-C)

Women:VAI = [Waist circumference (cm) / (36.58 + (1.88 × BMI))] × (Triglycerides / 0.81) × (1.52 / HDL-C)

Dietary inflammatory potential was assessed using the Dietary Inflammatory Index (DII), calculated according to the methodology developed by Shivappa et al., based on three-day dietary records. Dietary data were analyzed using the BEBIS software, which has been adapted and validated for use in Turkey [14].

Psychological assessments, including the Beck Depression Inventory, Beck Anxiety Inventory, and Epilepsy Stigma Scale, were administered by a neurologist and a psychiatrist. Eating behaviors were evaluated using the Three-Factor Eating Questionnaire. Calculations of the VAI and DII were performed by a registered dietitian.

To examine the complex interrelationships between dietary inflammatory potential, visceral adiposity, psychological characteristics, and eating behaviors, Structural Equation Modeling (SEM) was employed. SEM analyses were conducted using AMOS version 26.0, with latent variables constructed for psychological characteristics and eating behaviors, and observed variables including DII and VAI. Model fit was evaluated using standard indices, including the Comparative Fit Index (CFI), Tucker-Lewis Index (TLI), Root Mean Square Error of Approximation (RMSEA), and Standardized Root Mean Square Residual (SRMR). Direct, indirect, and total effects were estimated to assess the hypothesized pathways.

## 4 Results

The study population comprised 150 individuals with idiopathic generalized epilepsy and 140 control participants without a history of epilepsy. The mean age was 30.80 ± 10.46 years in the epilepsy group and 31.90 ± 10.52 years in the control group, with no significant difference between groups (p = 0.695). In the epilepsy group, 70 participants were female and 80 were male, while the control group included 65 females and 75 males (p = 0.063).

With respect to marital status, 30 of the 70 female and 45 of the 80 male participants with epilepsy were married. In the control group, 48 of the 65 female participants and 50 of the 75 male participants were married, with no significant group difference observed (p = 0.346). Educational status was also comparable between groups. In the epilepsy group, 50 of the 70 female participants and 20 of the 80 male participants had a high school education or below, whereas the remaining participants had higher education. In the control group, 40 of the 65 female participants and 25 of the 75 male participants had a high school education or below (p = 0.069).

Mean body mass index (BMI) was significantly higher in individuals with epilepsy compared with controls (24.5 ± 5.71 vs. 23.1 ± 7.36, p = 0.03). Among participants with epilepsy, 90 individuals (40 women and 50 men) were receiving polytherapy, while 60 individuals (30 women and 30 men) were receiving monotherapy. When stratified by epilepsy duration in five-year intervals, the polytherapy group included 20 individuals each in the 0–5, 10–15, and ≥15-year categories, and 30 individuals in the 5–10-year category. In the monotherapy group, 20 individuals were in each of the 0–5, 5–10, and 10–15-year categories, while 10 individuals had an epilepsy duration of ≥15 years.

Employment status differed significantly between groups. In the epilepsy group, 15 of the 70 female participants and 45 of the 80 male participants were actively employed, whereas in the control group, 30 of the 65 female participants and 60 of the 75 male participants were employed (p = 0.03).

According to the Beck Depression Inventory, depressive symptoms of varying severity were identified in 79 individuals with epilepsy and 40 individuals in the control group. In the epilepsy group, depressive symptoms were classified as minimal in 20 participants, mild in 15, moderate in 20, and severe in 24. In the control group, 20 participants had minimal symptoms, 10 had mild symptoms, 5 had moderate symptoms, and 5 had severe symptoms. Anxiety was detected in 50 individuals in the epilepsy group and 30 individuals in the control group. Among participants with epilepsy, anxiety severity was classified as severe in 18, moderate in 20, and mild in 12 individuals. In the control group, mild anxiety was observed in 19 participants and moderate anxiety in 11.

Among individuals with epilepsy, stigma scores were distributed as follows: among women, 10 reported mild stigma, 40 moderate stigma, and 20 severe stigma; among men, 25 reported mild stigma, 40 moderate stigma, and 15 severe stigma. No significant difference in stigma severity was observed between female and male participants (p = 0.09).

Evaluation of the Three-Factor Eating Questionnaire revealed that Individuals with epilepsy scored significantly higher than controls on both the emotional eating (18.88 ± 6.08 vs 10.66 ± 6.30, p = 0.001) and uncontrolled eating (24.44 ± 4.21 vs 17.58 ± 4.70, p = 0.002) subscales (Table 1).

**Table 1:** Comparison of three-factor eating scale scores.

	Epilepsy group $\overset{¯}{x}$± SS	Control group $\overset{¯}{x}$± SS	P value
Uncontrolled eating	24.44 ± 4.21	17.58 ± 4.70	**0.002**
Emotional eating	18.88 ± 6.08	10.66 ± 6.30	**0.001**
Cognitive restraint	11.29 ± 4.21	11.0 ± 4.70	0.062

Mann–Whitney *U* test.

Table 2 presents the mean scores of the Three-Factor Eating Questionnaire according to clinical and demographic characteristics within the epilepsy group. Significant differences were observed across all three eating behavior domains according to sex, employment status, marital status, educational level, epilepsy duration, treatment modality (mono- vs. polytherapy), and seizure frequency. Cognitive restraint scores were higher among female and single individuals with epilepsy and decreased with increasing epilepsy duration and seizure frequency. Emotional eating and uncontrolled eating scores were higher among women, unemployed individuals, married participants, and those receiving polytherapy. Both emotional eating and uncontrolled eating scores increased with longer disease duration and higher seizure frequency.

**Table 2:** Comparison of eating behavior scores according to clinical and demographic characteristics in individuals with epilepsy.

	Cognitive restraint* $\overset{¯}{x}$± SS	Emotional eating ** $\overset{¯}{x}$± SS	Uncontrolled eating *** $\overset{¯}{x}$± SS	p
Female (n: 70)	13.6 ± 6.08	16.6 ± 6.30	23.1 ± 6.66	P*= 0,034 P**= 0.028 P***= 0.002
Male (n:80)	10.3 ± 4.21	13.6 ± 6.30	16.3 ± 6.21	
Employee (n:60)	11.9 ± 5.05	17.9 ± 5.05	19.7 ± 6.67	P*= 0.044
Unemployed (n:90)	12.3 ± 4.58	18.3 ± 4.58	21.8 ± 6.14	P**= 0.038 P***= 0.042
Married (n: 75)	10.5 ± 4.25	17.8 ± 5.93	21.8 ± 6.14	P*= 0.021
Single (n: 75)	13.0 ± 4.93	11.3 ± 4.16	18.1 ± 5.18	P**= 0.019 P***= 0.033
Duration of epilepsy 0–5 years	13.3 ± 4.35	14.2 ± 6.51	19.6 ± 6.62	P*= 0.024 P**= 0.038 P***= 0.032
6–15 years	12.2 ± 4.71	17.8 ± 5.93	21.1 ± 5.35	
15 < years	9.7 ± 3.97	19.6 ± 4.75	23.2 ± 5.92	
Polytherapy (n:90)	11.1 ± 3.98	18.3 ± 5.76	25.7 ± 5.56	P*=0,093 P**=0.016 P***=0.003
Monotherapy (n:60)	11.1 ± 5.13	14.6 ± 6.12	21.2 ± 6.52	
Once or twice a year	11.9 ± 3.05	13.1 ± 5.67	19.7 ± 6.17	P*= 0.046 P**= 0.021 P***= 0.008
Once or twice a month	13.3 ± 4.58	19.6 ± 4.75	22.7 ± 3.73	
Frequency of seizures Once or twice a week	13.2 ± 4.71	20.2 ± 3.92	25.6 ± 3.14	

M, Mann Whitney *U* test, K, Kruskal Wallis test p < 0.05 VAI, Visceral Adiposity Index.

Correlation analyses demonstrated that depression, anxiety, and stigma scores were positively associated with emotional eating and uncontrolled eating, while cognitive restraint showed negative correlations with these psychological measures (Table 3).

**Table 3:** The correlation between individuals' eating behavior and various characteristics.

	Cognitive restraint		Emotional Eating		Uncontrolled Eating	
	r	p	r	p	r	p
Beck Anxiety	−0.271	**0.004**	0.470	**<0.001**	0.548	**<0.001**
Beck Depression	−0.247	**0.002**	0.559	**<0.001**	0.562	**<0.001**
Epilepsy Stigma Scale	−0.274	**0.004**	0.553	**<0.001**	0.219	**0.021**

Bivariate correlation p < 0.05.

The mean VAI score was 2.369 ± 1.364 in the epilepsy group and 2.341 ± 1.846 in the control group. Although this difference did not reach statistical significance, the mean VAI value was numerically higher in individuals with epilepsy. Age-specific cutoff values were applied to evaluate adipose tissue dysfunction. While no overt cardiovascular risk was identified, VAI values were positively correlated with epilepsy duration, stigma scores, emotional eating, and uncontrolled eating, and negatively correlated with cognitive restraint. Within the epilepsy group, VAI values were higher in men than in women (p = 0.004).

The mean VAI score among women with epilepsy was 2.012 ± 1.617, whereas the mean score among men was 2.407 ± 1.893. When analyzed by marital status, the mean VAI score was 2.345 ± 1.836 in married individuals and 2.302 ± 2.577 in single individuals; although not statistically significant, VAI values were higher among married participants (p > 0.05). Correlations between VAI values and epilepsy-related variables and eating behavior subscales are summarized in Table 4.

**Table 4:** The correlation of individuals' VAI value with the characteristics of various individuals with epilepsy.

	VAI	
	r	p
Cognitive restraint	−0.17	**0.038**
Emotional eating	0.165	**0.024**
Uncontrolled eating	0.219	**0.021**
Duration of epilepsy	0.282	**<0.001**
Epilepsy Stigma Scale	0.307	**<0.001**

Bivariate correlation *p < 0.05 VAI, Visceral Adiposity Index.

Mean DII scores were significantly higher in individuals with epilepsy compared with controls (1.53 ± 5.01 vs 0.00 ± 6.18, p = 0.035) (Table 5). Within the epilepsy group, higher DII scores were observed in individuals experiencing seizures once or twice per week compared with those experiencing seizures once or twice per year, and in those receiving polytherapy compared with monotherapy (Table 6).

**Table 5:** Comparison of Dietary Inflammatory Index (DII) between individuals with epilepsy and controls.

	Dietary inflammatory index			
	$\overset{¯}{x}$± SS	Median (lower–upper)	Test value	p
Individuals with epilepsy	1.53 ± 5.01	2.33 (−20.90–11.08)	−2.124	0.035
Control group	0.00 ± 6.18	1.11 (−19.63–10.43)		

Mann–Whitney *U* test.

**Table 6:** Comparison of DII according to seizure frequency and treatment modality in individuals with epilepsy.

	Dietary inflammatory index		
	$\overset{¯}{x}$± SS	Median (lower–upper)	p value
Frequency of seizures Once or twice a week	1.81 ± 5.03	2.46 (−20.90–11.08)	0.035
Once or twice a year	1.05 ± 4.31	1.86 (−20.90–11.08)	
Polytherapy	1.71 ± 4.78	2.26 (−13.94–9.95)	0.011
Monotherapy	0.83 ± 4.56	1.38 (−9.00–9.11)	

Kruskal Wallis.

In the epilepsy group, emotional eating and uncontrolled eating scores, VAI, and seizure frequency were positively correlated with DII, whereas cognitive restraint scores were negatively correlated with DII. In addition, seizure frequency differed significantly according to DII levels (ANOVA, p = 0.021) (Table 7).

**Table 7:** Correlation of Dietary Inflammatory Index with eating behaviors, psychological variables, and visceral adiposity.

Dietary inflammatory index		
	r	P value
Emotional eating	0.191	**0.002**
Uncontrolled eating	0.133	**0.037**
Cognitive restraint	−0.253	**0.000**
VAI	0.170	**0.007**
Beck depression	0.229	**<0.001**
Beck anxiety	0.204	**<0.001**
	Statistic	p
Frequency of epileptic episodes	F = 4.450	**0.021**
t: independent samples *t*-test; F: one-way analysis of variance *p < 0.05 VAI, Visceral Adiposity Index		

### 4.1 Path analysis findings

To further elucidate the direct and indirect relationships among dietary inflammatory load, psychological factors, visceral adiposity, and seizure frequency, a path analysis was conducted in individuals with epilepsy (n = 150). The model demonstrated acceptable goodness-of-fit indices (χ^2^/df = 2.11; CFI = 0.93; TLI = 0.91; RMSEA = 0.067; SRMR = 0.052).

DII showed significant direct effects on depression (β = 0.23, p < 0.001), anxiety (β = 0.20, p < 0.001), emotional eating (β = 0.18, p = 0.004), and VAI (β = 0.16, p = 0.01). Depression (β = 0.48, p < 0.001) and anxiety (β = 0.32, p = 0.002) were significantly associated with emotional eating.

Emotional eating demonstrated a positive effect on VAI (β = 0.17, p = 0.02). VAI, in turn, was significantly associated with seizure frequency (β = 0.19, p = 0.01). DII also retained a significant direct association with seizure frequency (β = 0.18, p = 0.02).

Bootstrap analysis (5000 samples) indicated a significant indirect effect of DII on seizure frequency mediated by emotional eating and VAI (indirect β = 0.06, 95% CI: 0.02–0.11), suggesting partial mediation.

**Fig. 1:**
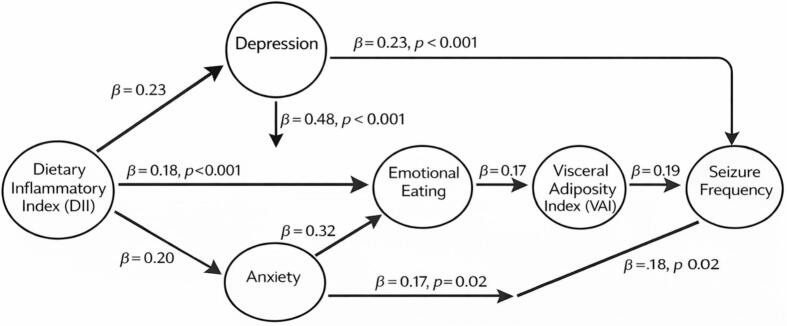
Path analysis model of dietary inflammatory load, psychological factors, visceral adiposity, and seizure frequency. Path analysis model illustrating the relationships among dietary inflammatory index (DII), psychological factors, emotional eating, visceral adiposity index (VAI), and seizure frequency in individuals with epilepsy. Standardized direct, indirect, and total effects derived from the path analysis are presented (see Table 8). DII was positively associated with depression (β = 0.23, p < 0.001), anxiety (β = 0.20, p < 0.001), emotional eating (β = 0.18, p = 0.004), and VAI (β = 0.16, p = 0.01). Depression (β = 0.48, p < 0.001) and anxiety (β = 0.32, p = 0.002) were significantly associated with emotional eating. Emotional eating was positively related to VAI (β = 0.17, p = 0.02), and VAI was associated with seizure frequency (β = 0.19, p = 0.01). DII exerted both a direct effect on seizure frequency (β = 0.18, p = 0.02) and an indirect effect mediated sequentially by emotional eating and VAI (β = 0.06), resulting in a significant total effect (β = 0.24, p < 0.001). Solid arrows represent direct paths, whereas dashed arrows indicate indirect pathways. *p < 0.05; **p < 0.01; ***p < 0.001.

The total effect of DII on seizure frequency remained significant (β = 0.24, p < 0.001), indicating that both direct and indirect pathways contributed to seizure burden. The standardized direct, indirect, and total effects obtained from the path analysis are presented in Table 8, and the overall structural model is illustrated in Fig. 1**.**

**Table 8:** Standardized Direct, Indirect, and Total Effects from Path Analysis.

Path	Standardized β	p-value
DII → Depression	0.23	<0.001
DII → Anxiety	0.20	<0.001
DII → Emotional Eating	0.18	0.004
DII → VAI	0.16	0.01
Depression → Emotional Eating	0.48	<0.001
Anxiety → Emotional Eating	0.32	0.002
Emotional Eating → VAI	0.17	0.02
VAI → Seizure Frequency	0.19	0.01
DII → Seizure Frequency (direct)	0.18	0.02
DII → Seizure Frequency (indirect)	0.06 (95% CI: 0.02–0.11)	
DII → Seizure Frequency (total)	0.24	<0.001

DII, Dietary Inflammatory Index; VAI, Visceral Adiposity Index.

Sex-based comparisons within the epilepsy group demonstrated that although women had higher eating behavior scale scores, VAI values were higher in men, while DII values were higher in women. These findings are presented in Table 9.

**Table 9:** Comparison of DII and VAI in epilepsy patients by gender.

	DII $\overset{¯}{x}$± SS	P (Student’s–*t* test)	VAI	P (Mann Whitney U)
Female	1.77 ± 2.63	**0.001**	2.012 ± 1.617	**0.004**
Male	1.02 ± 1.91		2.407 ± 1.893	

DII, Dietary Inflammatory Index; VAI, Visceral Adiposity Index.

## 5 Discussion

To our knowledge, this study is the first to simultaneously evaluate the Visceral Adiposity Index (VAI) and the Dietary Inflammatory Index (DII) in individuals with epilepsy. The findings suggest that pro-inflammatory dietary patterns and increased visceral adiposity may be associated with heightened cardiometabolic risk, particularly among patients with epilepsy who also experience psychological comorbidities such as anxiety, depression, and perceived stigma.

Despite substantial advances in antiseizure pharmacotherapy, optimal seizure control remains elusive for a considerable proportion of patients, underscoring the importance of modifiable lifestyle factors, including dietary habits [15]. Accumulating evidence indicates that inflammatory mechanisms play a central role in both the initiation and progression of epilepsy [16]. Systemic inflammation may compromise blood–brain barrier integrity, thereby facilitating the translocation of circulating cytokines and immune cells into the central nervous system and promoting neuroinflammatory processes that contribute to epileptogenesis [17], [18], [19].

In the present study, individuals with more frequent seizures and those receiving polytherapy exhibited higher DII scores, reflecting greater adherence to pro-inflammatory dietary patterns. This observation suggests that dietary inflammatory load may be linked to disease severity and less favorable clinical outcomes. Consistent with previous research, specific dietary components and overall dietary patterns have been shown to exert substantial effects on systemic inflammatory responses [20].

Dietary patterns rich in antioxidants have been associated with reduced epilepsy risk and improved psychiatric outcomes, including lower levels of depression and anxiety [21], [22], [23], [24], [25]. In line with these findings, we observed positive correlations between DII scores and both depression and anxiety measures, supporting the hypothesis that diet-related inflammation may represent a shared biological pathway connecting epilepsy and psychological distress.

Pro-inflammatory diets are known to elevate systemic inflammatory burden [26]. A comprehensive review published in 2025 identified inflammation as one of the most common etiological factors in drug-resistant epilepsy and highlighted the role of multiple inflammatory markers in this process [27]. Systemic inflammation may contribute to epileptogenesis by enabling circulating inflammatory mediators to cross a compromised blood–brain barrier and induce neuroinflammatory cascades [28]. In addition, circulating monocytes can migrate into the brain under inflammatory conditions and differentiate into macrophage- or microglia-like cells, further amplifying neuroinflammation [29]. The path analysis provided further support for the complex interplay between dietary inflammation, psychological distress, visceral adiposity, and seizure burden. Importantly, DII exerted both direct and indirect effects on seizure frequency, partially mediated by emotional eating and visceral adiposity. These findings suggest that inflammatory dietary patterns may influence epilepsy severity not only through systemic metabolic pathways but also via psychological mechanisms. In addition, the path model indicated that visceral adiposity also contributed to seizure burden, suggesting that metabolic alterations related to visceral fat accumulation may represent an additional pathway linking dietary inflammation and epilepsy severity.

Diet also plays a pivotal role in shaping the gut microbiota, often referred to as the “second brain,” which exerts substantial influence on immune regulation and neural signaling [30]. Experimental studies have demonstrated that stress-induced alterations in gut microbiota composition may increase seizure susceptibility. Moreover, gut dysbiosis has been implicated in the pathophysiology of drug-resistant epilepsy, and probiotic supplementation has been associated with reductions in seizure frequency and improvements in quality of life [31], [32], [33]. These observations support the plausibility that the DII may influence epilepsy not only through systemic inflammatory pathways but also via mechanisms involving the gut–brain axis.

The VAI, which integrates waist circumference, BMI, and metabolic parameters, serves as a sex-specific indicator of visceral adipose tissue dysfunction and cardiometabolic risk. Compared with conventional anthropometric indices, VAI has demonstrated greater sensitivity in reflecting metabolic and inflammatory risk profiles [34], [35]. Previous studies have reported close associations between VAI and systemic inflammation, with inflammatory indices correlating positively with visceral adiposity. In agreement with this literature, we observed a positive correlation between DII and VAI in individuals with epilepsy. Visceral obesity is characterized by increased secretion of adipocytokines and pro-inflammatory cytokines, thereby contributing to a state of chronic low-grade inflammation [36], [37], [38]. Notably, a study published in 2025 demonstrated sex-specific alterations in peripheral immune function and cytokine profiles among individuals with drug-resistant epilepsy [39].

Consistent with these findings, our sex-based analyses revealed higher DII values in women and higher VAI values in men, despite a higher prevalence of maladaptive eating behaviors among women. These results underscore the importance of considering sex-specific metabolic and inflammatory differences when evaluating dietary and body composition–related factors in epilepsy.

Overall, the findings of this study indicate that nutritional factors and inflammation play a meaningful role in the clinical course of epilepsy and may contribute to both metabolic risk and disease severity.

## 6 Limitations

Several limitations of this study should be acknowledged. First, the cross-sectional design precludes any causal inference regarding the observed associations among dietary inflammatory load, visceral adiposity, and epilepsy-related clinical and psychological variables. Second, the single-center nature of the study limits the generalizability of the findings to more diverse populations.

Dietary intake was assessed using three-day food records, which may not fully capture habitual dietary patterns and are inherently susceptible to recall bias. Psychological status and eating behaviors were evaluated using self-reported questionnaires, introducing the potential for reporting and measurement bias. In addition, several important confounding factors—including socioeconomic status, physical activity level, and sleep characteristics—were not comprehensively assessed or controlled.

The potential metabolic and inflammatory effects of antiseizure medications, particularly in individuals receiving polytherapy, could not be isolated. Although we stratified participants according to monotherapy versus polytherapy and examined associations with the DII in both groups, the sample size did not allow for robust evaluation of individual antiseizure medications’ specific effects on inflammatory and metabolic outcomes. Therefore, potential drug-specific influences remain to be elucidated in future studies. Finally, the absence of laboratory-based inflammatory biomarkers limits the ability to draw mechanistic conclusions regarding the pathways linking diet, inflammation, and epilepsy.

## 7 Conclusion

This study demonstrates that eating behaviors, dietary inflammatory load, and visceral adiposity are closely associated with key clinical and psychological features of epilepsy, including seizure frequency, disease duration, treatment modality, and psychiatric comorbidities. Individuals with epilepsy exhibited more pro-inflammatory dietary patterns, higher levels of emotional and uncontrolled eating, and greater visceral adiposity, all of which were linked to increased depression, anxiety, and perceived stigma.

These findings suggest that inflammation-related nutritional factors may play a meaningful role in both the clinical course of epilepsy and its associated metabolic risk profile. Given the complex and potentially bidirectional interactions among diet, systemic inflammation, neuroinflammatory processes, and seizure susceptibility, integrating nutritional assessment and targeted dietary interventions into routine epilepsy care may provide important complementary benefits.

Future longitudinal and mechanistic studies are warranted to clarify causal relationships and to determine whether anti-inflammatory or microbiota-modulating dietary strategies can improve seizure control, psychological well-being, and overall health outcomes in individuals with epilepsy.

## Ethical approval and consent to participate

This study was approved by the Hitit University School of Medicine Ethics Committee () and conducted by following STROBE guidelines for reporting observational studies. The study was approved by the institutional ethics review board and complied with the Declaration of Helsinki. All participants gave their informed consent for this study.

Consent for publication

All authors gave their informed consent for publication of the article. Detailed consent was obtained from each individual participating in the study.

## CRediT authorship contribution statement

**Sinan Eliaçık:** Writing – review & editing, Writing – original draft, Visualization, Validation, Supervision, Software, Resources, Project administration, Methodology, Investigation, Funding acquisition, Formal analysis, Data curation, Conceptualization. **Sena Yanıkoğlu:** Writing – review & editing, Supervision, Methodology, Data curation. **Ayşe Erdoğan Kaya:** Writing – review & editing, Resources, Methodology, Formal analysis, Conceptualization.

## Funding

No funding was received for this research.

## Declaration of competing interest

The authors declare that they have no known competing financial interests or personal relationships that could have appeared to influence the work reported in this paper.

## Data Availability

The authors confirm that the data supporting the findings of this study are available within the article [and/or] its supplementary materials.

## References

[1] Kalnina I., Straumite E., Klava D., Kruma Z., Bartkiene E., Isoldi K.K. (2022). Analysis of factors that influence eating habits in different countries. J Hygienic Eng Design.

[2] Ünsal A. (2019). Beslenmenin önemi ve Temel Besin Öğeleri. Kırşehir Ahi Evran Üniversitesi Sağlık Bilimleri Dergisi.

[3] Borowicz-Reutt K., Krawczyk M., Czernia J. (2024). Ketogenic diet in the treatment of epilepsy. Nutrients.

[4] Özkan Y. (2017). Ergenlerde Sosyal Görünüş Kaygısı ile Duygusal Yeme Arasındaki İlişkinin İncelenmesi (Master's Thesis. İstanbul Gelişim Üniversitesi Sosyal Bilimler Enstitüsü).

[5] İnalkaç S., Arslantaş H. (2018). Duygusal Yeme. Aktd.

[6] Evers C., Dingemans A., Junghans A.F., Boevé A. (2018). Feeling bad or feeling good, does emotion affect self-control?. Psychol Sci.

[7] Ayyıldız F., Ülker İ., Yıldıran H. (2021). Reflection of hedonic hunger and eating behavior relationship on different body masses. J Nutrition Diet.

[8] Kocamış R.N. (2018).

[9] Bodur M., Ünal R.N. (2019). Kronik hastalıklar ekseninde diyette yüksek fruktoz ve doymuş yağ asitlerinin kronik düşük derece inflamasyon üzerine etkisi. Cukurova Med J.

[10] Yıldırım Çavak B., Andaç Ö.S. (2020). Postmenopozal Kadınlarda Diyet İnflamatuar İndeksi ve Kırık İnsidansı İlişkisi. İZÜFBED.

[11] Seremet Kürklü N., Torun N.K., Özen Küçükçetin I., Akyol A. (2020). Is there a relationship between the dietary inflammatory index and metabolic syndrome among adolescents?. J Pediatr Endocrinol Metab.

[12] Jakubiak G.K., Badicu G., Surma S., Waluga-Kozłowska E., Chwalba A., Pawlas N. (2025). The visceral adiposity index and its usefulness in the prediction of cardiometabolic disorders. Nutrients.

[13] Amato M.C., Giordano C., Galia M., Criscimanna A., Vitabile S., Midiri M. (2010). AlkaMeSy Study Group. visceral adiposity index: a reliable indicator of visceral fat function associated with cardiometabolic risk. Diabetes Care.

[14] Shivappa N., Steck S.E., Hurley T.G., Hussey J.R., Hébert J.R. (2013). Designing and developing a literature-derived, populationbased dietary inflammatory index. Public Health Nutr.

[15] Radzik I., Miziak B., Dudka J., Chroscinska-Krawczyk M., Czuczwar S.J. (2015). Prospects of epileptogenesis prevention. Pharmacol Rep.

[16] Sun H., Ma D., Hou S., Zhang W., Li J., Zhao W. (2024). Exploring causal correlations between systemic inflammatory cytokines and epilepsy: a bidirectional Mendelian randomization study. Seizure.

[17] Wang A., Si Z., Li X., Lu L., Pan Y., Liu J. (2019). FK506 attenuated pilocarpine-induced epilepsy by reducing inflammation in rats. Front Neurol.

[18] Stredny C., Rotenberg A., Leviton A., Loddenkemper T. (2023). Systemic inflammation as a biomarker of seizure propensity and a target for treatment to reduce seizure propensity. Epilepsia Open.

[19] Rana A., Musto A.E. (2018). The role of inflammation in the development of epilepsy. J Neuroinflammation.

[20] Hart M.J., Torres S.J., McNaughton S.A., Milte C.M. (2021). Dietary patterns and associations with biomarkers of inflammation in adults: a systematic review of observational studies. Nutr J.

[21] Ding R., Han Z., Gui J., Xie L., Yang J., Yang X. (2023). Inflammatory properties of diet mediate the effect of epilepsy on moderate to severe depression: results from NHANES 2013-2018. J Affect Disord.

[22] Zhang Y., Shen J., Su H., Lin C. (2024). Association between composite dietary antioxidant index and epilepsy in American population: a cross-sectional study from NHANES. BMC Public Health.

[23] He X., Li Z., Wu H., Wang L., Zhang Y. (2024). Composite dietary antioxidant index mediates the effect of epilepsy on psychiatric disorders: results from NHANES 2013-2018. Front Neurol.

[24] Park J., Jeong K.H., Shin W.H., Bae Y.S., Jung U.J., Kim S.R. (2016). Naringenin ameliorates kainic acid-induced morphological alterations in the dentate gyrus in a mouse model of temporal lobe epilepsy. Neuroreport.

[25] Liang Z., Lou Y., Zheng Z., Guo Q., Liu S. (2024). Diet-derived circulating antioxidants and risk of epilepsy: a study combining metabolomics and mendelian randomization. Heliyon.

[26] D'Esposito V., Di Tolla M.F., Lecce M., Cavalli F., Libutti M., Misso S. (2022). Lifestyle and dietary habits affect plasma levels of specific cytokines in healthy subjects. Front Nutr.

[27] Pinzon-Hoyos N., Li Y., McGee M., Poolos N., Marchi N., Brewster A.L. (2025). Drug-resistant epilepsy associated with peripheral complement decreases and sex-specific cytokine imbalances: a pilot study. Sci Rep.

[28] Sun Y., Koyama Y., Shimada S. (2022). Inflammation from peripheral organs to the brain: how does systemic inflammation cause neuroinflammation?. Front Aging Neurosci.

[29] Li W., Wu J., Zeng Y., Zheng W. (2023). Neuroinflammation in epileptogenesis: from pathophysiology to therapeutic strategies. Front Immunol.

[30] Makki K., Deehan E.C., Walter J., Backhed F. (2018). The impact of dietary Fiber on gut microbiota in host health and disease. Cell Host Microbe.

[31] Medel-Matus J.S., Shin D., Dorfman E., Sankar R., Mazarati A. (2018). Facilitation of kindling epileptogenesis by chronic stress may be mediated by intestinal microbiome. Epilepsia Open.

[32] Peng A., Qiu X., Lai W., Li W., Zhang L., Zhu X. (2018). Altered composition of the gut microbiome in patients with drug-resistant epilepsy. Epilepsy Res.

[33] Gomez-Eguilaz M., Ramon-Trapero J., Perez-Martinez L., Blanco J.R. (2018). The beneficial effect of probiotics as a supplementary treatment in drug-resistant epilepsy: a pilot study. Benef Microbes.

[34] Bozorgmanesh M., Sardarinia M. (2016). Cvd-Predictive performances of a body shape index versus simple anthropometric measures: Tehran lipid and glucose study. Eur J Nutr.

[35] Moreno C., Tandon R. (2011). Should overeating and obesity be classified as an addictive disorder in Dsm-5. Curr Pharm Des.

[36] Karabay E., Karşıyakalı N., Kayar K., Verim L., Tosun Ç., Yücebaş Ö. (2020). Evaluation of relationship between visceral adiposity ındex and overactivate bladder symptoms in females. Endoüroloji Bülteni.

[37] Liu X., Zhang Y., Li Y., Sang Y., Chai Y., Zhang L. (2024). Systemic immunity-inflammation index is associated with body fat distribution among U.S. adults: evidence from national health and nutrition examination survey 2011-2018. BMC Endocr Disord.

[38] Nameni G., Jazayeri S., Salehi M., Esrafili A., Hajebi A., Motevalian S.A. (2024 Jan 25). Association between visceral adiposity and generalized anxiety disorder. BMC Psychol.

[39] Hinojosa-Figueroa M.S., Cruz-Caraguay M., Torres Pasquel A., Puga Rosero V., Eguiguren Chavez C.B., Rodas J.A. (2025). Etiologies of multidrug-resistant epilepsy in Latin America: a comprehensive review of structural, genetic, metabolic, inflammatory, and infectious origins: a systematic review. Biomolecules.

